# Effects of Light-Burnt Dolomite Incorporation on the Setting, Strength, and Drying Shrinkage of One-Part Alkali-Activated Slag Cement

**DOI:** 10.3390/ma12182874

**Published:** 2019-09-05

**Authors:** In Kyu Jeon, Jae Suk Ryou, Sadam Hussain Jakhrani, Hong Gi Kim

**Affiliations:** Department of Civil and Environmental Engineering, Hanyang University, Seoul 04763, Korea (I.K.J.) (J.S.R.) (S.H.J.)

**Keywords:** light-burnt dolomite, GGBFS, setting, strength, drying shrinkage, mortar

## Abstract

This study investigates the potential of light-burnt dolomite (LBD) as a supplementary cementitious material with ground granulated blast furnace slag (GGBFS) and Ordinary Portland cement (OPC). In this work, LBD was substituted for up to 20% of GGBFS in sodium sulfate-activated slag systems. The effects of LBD incorporation on the flow, setting time, compressive and flexural strength development, and drying shrinkage were explored with, X-ray diffraction and thermogravimetric analyses. LBD incorporation resulted in greater strength development of an alkali-activated slag system. The optimum LBD content for strength development was 10%, regardless of ordinary Portland cement content. In addition, LBD decreased the drying shrinkage, accelerated the hydration process, and induced hydrotalcite formation, which can be attributed to the reactive MgO inside LBD.

## 1. Introduction

Ordinary Portland cement (OPC) is a common raw material for concrete structures. However, the cement manufacturing process is considered to be a major factor in global warming and air pollution because it emits a large amount of carbon dioxide [1]. In alkali-activated cement (AAC), all or part of the cement binder is replaced with supplementary cementitious materials (SCM); thus, AAC is a potential eco-friendly alternative that can reduce carbon dioxide emissions [2,3]. AAC is generally obtained from two-part reactions with solid aluminosilicate precursors and concentrated aqueous alkali activators [4]. However, two-part reactive AAC with corrosive and viscous alkali activators, such as sodium silicate solution has high costs, due to handling and application problems [5,6]. Recent studies have focused on the development of user-friendly one-part AAC, in which only water is added [7,8,9]. 

Studies have recently been conducted on alkali-activated slag (AAS), which is a type of AAC that is an alternative to OPC [10,11,12]. Slag has a similar chemical composition to OPC, and several studies indicate that AAS has good strength development with lower calcium content [13,14]. In AAS procedures, the alkali activation mechanism consists of the combined reaction of destruction-condensation, where the primary material is destroyed in low stable structural units and interacted with condensed structure. As a result of this reaction, low crystalline calcium silicate hydrate (C-S-H) with a low CaO/SiO_2_ ratio is produced in the activation process [15,16]. AAS has different chain structures than OPC: While, OPC consists of linear finite chains with SiQ^1^ and SiQ^2^, AAS has a long chain organized with SiQ^2^ or SiQ^2^ (1Al) [17]. 

Previous studies have attempted to enhance the mechanical properties of AAS by incorporating different admixtures. Chang et al. showed that incorporating gypsum in AAS pastes increased the compressive strength and decreased the setting time. Park et al. demonstrated that AAS activated by clinker-free CaO, substituted with gypsum, had positive effects on strength development, due to ettringite formation. 

However, AAS has larger shrinkage values than OPC, which has led to a decrease in its use [18,19]. Previous research [20,21] found that the drying shrinkage of AAS was 2 to 3 times higher than OPC under the same curing environment. Thomas et al. [22] indicated that capillary stress, surface tension, and disassembling pressure were the major factors in the drying shrinkage of cementitious materials. Ye et al. [23,24] found that the structural combinations of alkali cations, in C-S-H and C-A-S-H, which led to a diminished stacking uniformity of C-S-H and C-A-S-H, also made it easier to collapse and repartition against drying conditions. To decrease the negative effects of shrinkage in an AAS system, Neto et al. [25] showed that the presence of sodium silicate and silica in AAS decreased shrinkage, due to additional ettringite formation. 

Dolomite [CaMg(CO_3_)_2_] is obtained from mineral dolomite rock, consisting of calcium, magnesium, and carbonate. In high-alkaline conditions, dolomite undergoes a de-dolomitization reaction, reacting with portlandite to produce calcite and brucite [26,27]. The light-burnt dolomite (LBD) is produced by raw dolomite through a calcination process at 900–1000 °C and consists mainly of reactive CaO and MgO. LBD is an economical powder-phased lime material that is used as an activator. A previous study [28] used LBD, up to 2%, as an activator with sodium silicate solution, in order to analyze the hydration and strength development of AAS. However, other studies have not used LBD as an SCM. If LBD could be activated like GGBFS and shows higher, not only mechanical properties, but shrinkage compensate performance, than the GGBFS mixture, the feasibility of using LBD as SCM will be demonstrated. Additionally, large amounts of reactive MgO can play a role in mechanical and durability performance of cement composite [29]. Therefore, this study aimed to investigate the influence of LBD used as a SCM on the hydration process, strength development, and drying shrinkage of AAS. For this purpose, workability, compressive and flexural strength, isothermal conduction calorimetry (ICC), and drying shrinkage were explored with X-ray diffraction analysis (XRD) and thermogravimetric analysis (TGA).

## 2. Experimental Process

### 2.1. Materials

**Figure 1:**
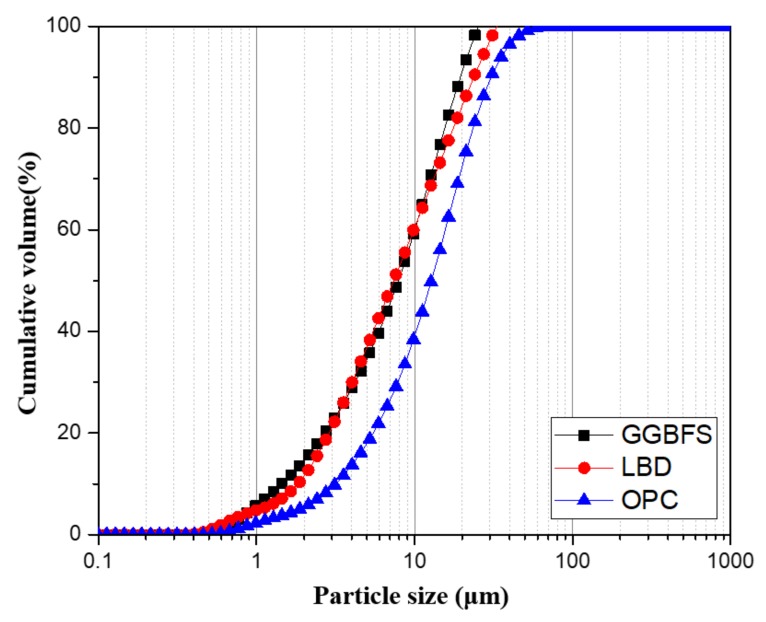
Particle size distributions of Ordinary Portland cement (OPC), ground granulated blast furnace slag (GGBFS), and light-burnt dolomite (LBD).

**Figure 2:**
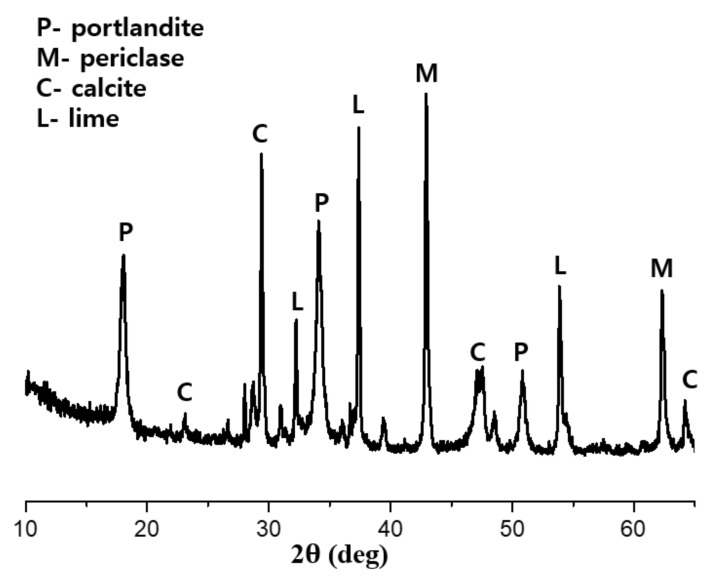
XRD patterns of LBD.

OPC complying with ASTM C150 [30], ground granulated blast furnace slag (GGBFS), and light-burnt dolomite (LBD), obtained from a local R company in Gwangyang, Republic of Korea were used as binders in this study. Sodium sulfate anhydrous with a purity of 99% was used in this study as an activator. The chemical and physical properties of the OPC, GGBFS, and LBD were determined with X-ray fluorescence (XRF) (S8 Tiger, Bruker, Billerica, MA, USA), as presented in Table 1. Figure 1 shows the particle size distributions of OPC, GGBFS, and LBD, which were measured with a particle size analyzer (Malvern Mastersizer 2000, Malvern, Malvern, England). The specific surface area of the raw materials was obtained from BET method using a surface area analyzer (3 Flex, micromeritics instruments, Micromeritics, GA, USA). Figure 2 shows the mineralogical composition of LBD, obtained from XRD analysis. Natural sand with a maximum size of 5 mm, absorption of 1.05%, and fineness modulus (F.M) of 2.7 was used as a fine aggregate in this study. 

### 2.2. Mix Proportions and Fabrication of Mortar Specimens

The mortar mix proportions used in this study are presented in Table 2. The mortar mixture had a fine aggregate-to-binder weight ratio of 2 and a water-to-binder weight ratio of 0.4. Sodium sulfate is an alkali activator, with 4% of the binder weight used. The amount of cement in the mixtures was fixed at 10% or 20%. LBD was replaced (by weight) with 0%, 10%, and 20% of GGBFS. The values are denoted as C10S90, C10S80L10, C10S70L20, C20S80, C20S70L10, and C20S60L20. For example, C10S80L10 indicates a mortar mix made with 10% cement, 80% GGBFS, and 10% LBD.

Mortar cubes (50 × 50 × 50 mm^3^) and prisms (40 × 40 × 160 mm^3^) were prepared in accordance with ASTM C109 [31], and ASTM C348 [32], respectively. After mixing, the cube and prisms molds were vibrated for 30 s using a vibration table to remove air voids. Mortar samples were then sealed in plastic wrap to prevent moisture evaporation and stored in ambient conditions (20 ± 5 °C and 60 ± 5% relative humidity) for 24 h. After 24 h, the mortar samples were demolded and kept in water at 23 ± 2 °C for curing until the desired duration was reached.

### 2.3. Testing Methods

In this study, the properties of fresh mortar mixtures were determined by testing their workability. The workability of mixtures was tested using the flow table according to ASTM C230 (ASTM, 2004b) [33], and the procedure followed ASTM C1437 (ASTM, 2001) [34]. To observe the setting time of fresh mortar, the initial and final setting times were investigated using Vicat needles in accordance with ASTM C191 (ASTM, 2004a) [35].

To evaluate the mechanical properties of AAS, the compressive strengths of cube samples were measured using a universal testing machine (Shimadzu, CCM-200A; Shimadzu Corporation, Kyoto, Japan), in accordance with ASTM C109 [31]. A flexural strength test was conducted on the prism samples, according to the ASTM C348 [36] after 3, 7, and 28 days of water curing. For each test, three replicates were measured; the average value is presented. The heat evolution was investigated using a semi-adiabatic calorimeter according to a previous study [37]. For XRD and TGA analysis, powder specimens were obtained from the AAS mixtures after 7 days of hydration using an X-ray diffractometer (RINT D/max 2500, 40 kV, 30 Ma, scanning speed 2°/min, wavelength 1.54 Å) and a thermogravimetric analyzer (TGA7 PERKINELMER, TA instruments, PerkinElmer, Waltham, MA, USA). The drying shrinkage of AAS mixtures was tested using a dial gauge in accordance with ASTM 490 [38].

## 3. Results and Discussion

### 3.1. Flow Test

**Figure 3:**
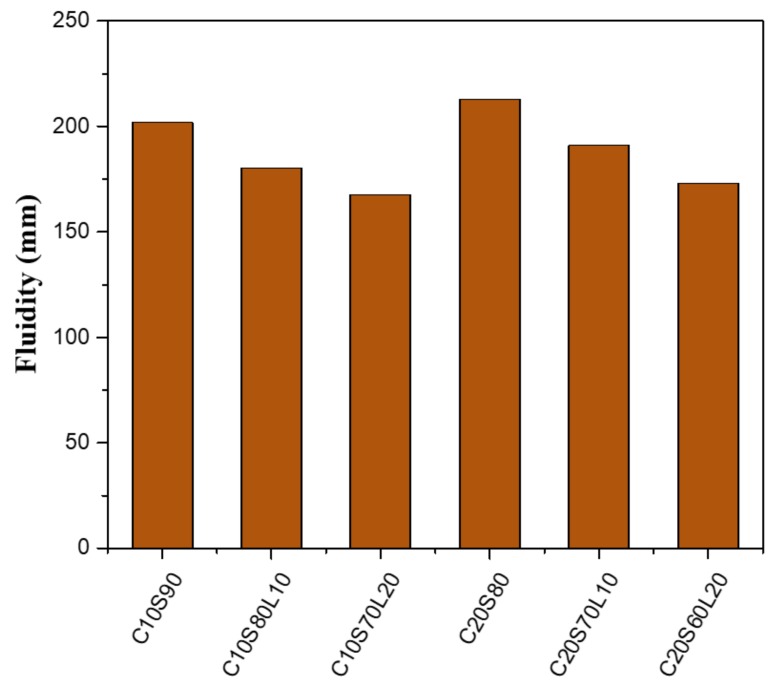
Fluidity of alkali-activated slag mixture.

Figure 3 shows the flow diameters of alkali-activated slag mortar mixtures. The flow value of AAS mortar was influenced by the replacement levels of LBD and OPC in the binder. For the OPC replacement levels, a reduction of OPC content in the mixture enhanced the GGBFS content and decreased the fluidity value, which is a similar trend to that seen in a previous paper [39,40]. The fluidity of C10S90, C10S80L10, and C10S70L20 decreased by 5.4%, 6.2%, and 3.1%, respectively, compared with C20S80, C20S70L10, and C20S60L20. Regardless of OPC content, an increasing amount of LBD led to a reduction of fluidity for all mortar mixes. In a mixture containing 10% OPC, the fluidity of C10S80L10 and C10S70L20 declined by approximately 10.7%, and 17%, respectively, compared with C10S90. With increasing replacement levels of LBD, a higher fluidity reduction effect appeared. The decrease in flow value is similar to the results reported in previous studies [41]. H. Ye et al. indicated that the dolomite incorporation decreases the workability of AAS, thus, leading to increased water demands to achieve a similar flow value. 

### 3.2. Setting Time

**Figure 4:**
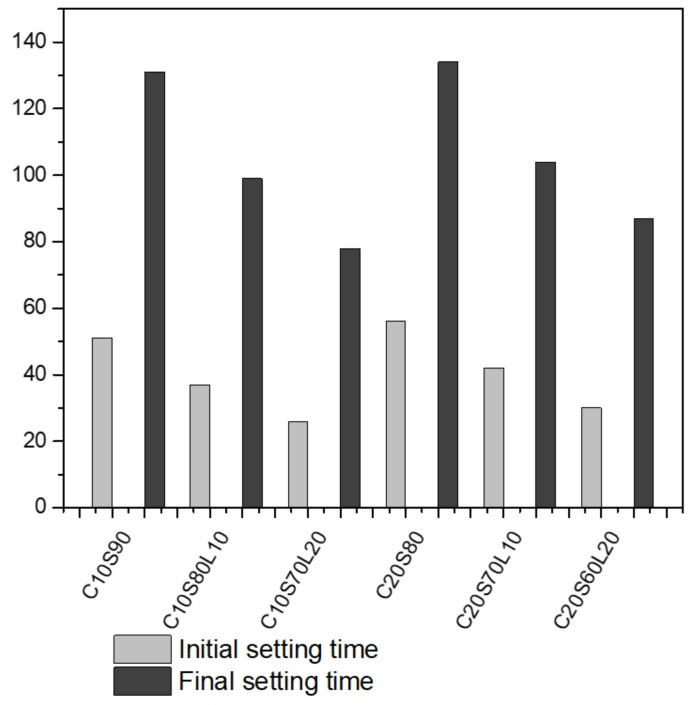
Initial and final setting time of alkali-activated slag (AAS) mixtures.

Figure 4 illustrates the initial and final setting times of an AAS mixture, incorporated with LBD. Setting time is an important factor in the strength development of cementitious materials, which normally occurs in a short time [19]. Generally, an AAS system has shorter initial and final setting times than OPC-based mixtures. In comparing 10% OPC mixture with 20% OPC mixture, the 20% OPC-based AAS showed slightly longer initial and final setting times, due to the amount of GGBFS content. Less OPC content led to increased GGBFS content in the same amount of binder; greater GGBFS content also decreased the setting time [42]. In addition, both the initial and final setting times decreased with enhanced LBD content. When the LBD content was increased from 10% to 20% in a 10% OPC-based AAS system, the initial setting values decreased by 37.8% and 96.15%, and the final setting values decreased by 32.3% and 67.9%, compared with only OPC, and a GGBFS-based AAS system, respectively. A 20% OPC-based AAS system has similar tendencies. When LBD increased from 10% to 20%, the initial setting values decreased by 33.3% and 86.6%, and the final setting values decreased by 28.8%, and 54%, respectively. The setting time can be affected by the generation heat release of materials. LBD has more reactivity than GGBFS and produces more heat release. The cumulative heat release of different LBD content in an AAS system is discussed in Section 3.3.

### 3.3. Isothermal Heat Release

**Figure 5:**
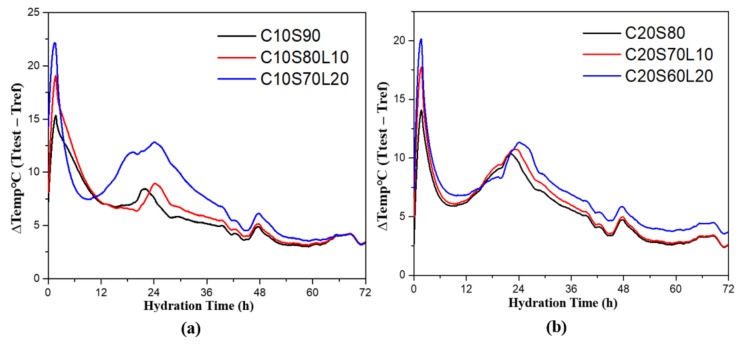
Calorimetric curves for the rate of heat release for (**a**) 10%, and (**b**) 20% OPC-containing AAS mixtures.

Generally, the effects of temperature on reaction kinetics were investigated using isothermal calorimetry. Figure 5 shows the calorimetric curves of the heat release of AAS mixtures. Figure 5a shows the heat release curve of 10% OPC-containing mixtures, and Figure 5b represents the heat release curve of 20% OPC-containing mixtures, which both have two distinct peaks. The first sharp peak occurred immediately after mixing all the mixtures. Bernal et al. [43] believed that this initial peak was produced from the dissolution of the dry mixture. With increasing LBD content, the samples showed higher maximum heat evolution rates for the dissolution peaks compared with 0% LBD samples. Thus, a higher LBD content promotes dissolution. A similar heat evolution enhancement was observed in a previous study of an AAS system with dolomite [28,44]. Yang et al. [28] found that the increase in heat evolution with LBD was due to the hydration procedure for LBD. Reactive CaO and MgO inside LBD, produced calcium hydroxide, C-A-S-H gel, and magnesium hydroxide, which affected the heat evolution of the AAS system. Ye et al. [41] also reported that the replacement of slag with dolomite, increased the Na_2_O content and caused the slag to react at a more advanced level.

The second peak occurred between 20 and 30 h. Zhang et al. [45] found that the acceleration peaks are affected by the reorganization of the C-A-S-H gel phase, which is related to the polymerization degree of the binder. Figure 5a,b show a higher second peak value with increasing LBD content. In both mixtures, increasing the amount of LBD caused the peak time to be slightly delayed, due to the high MgO content of LBD. LBD reacted with water and produced magnesium hydroxide, which is an insoluble product. During this formation process, the dissolved Mg^2+^ led to a decrease of hydroxyl ions in the solution, then caused a reduction in the alkalinity of the mixture [46].

**Figure 6:**
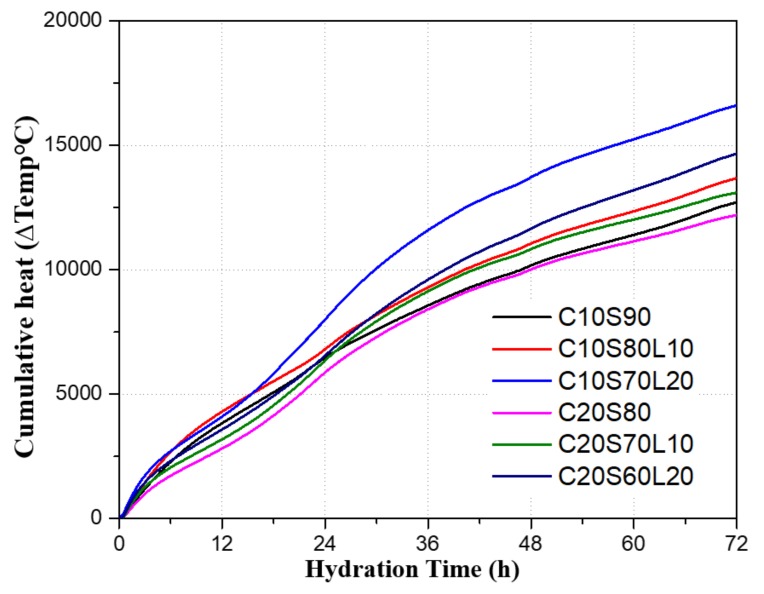
Cumulative heat release of AAS mixtures with different OPC and LBD content.

Figure 6 shows the cumulative heat release of AAS mixtures. The cumulative heat release sequence by mixture was as follows: C10S70L20, C20S60L20, C10S80L10, C20S70L10, C10S90, and C20S80. LBD incorporation enhanced the cumulative heat release compared to specimens without LBD. These increases in the cumulative heat release can affect the setting time, as was discussed in Section 3.2.

### 3.4. Compressive Strength

**Figure 7:**
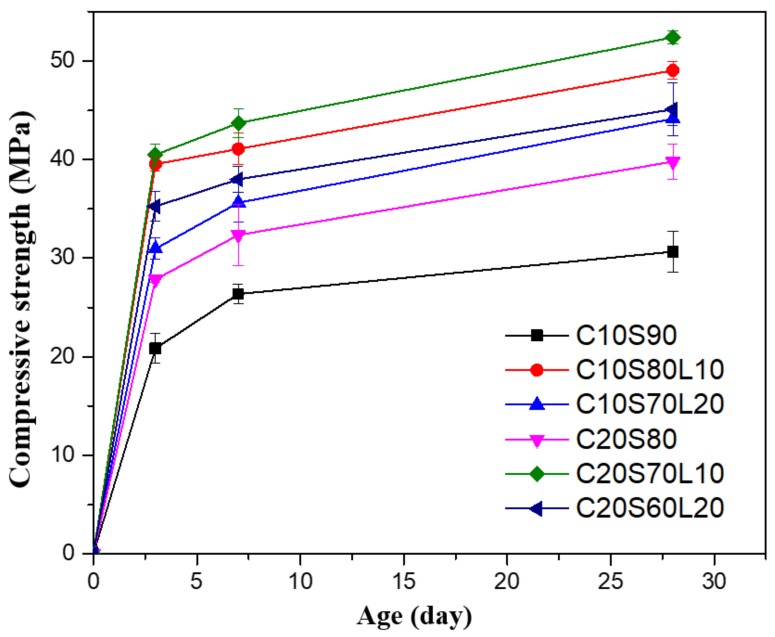
Compressive strength of alkali-activated slag mortar with different mixtures.

Figure 7 represents the compressive strength results of AAS specimens with different LBD and OPC replacement levels. The compressive strength development showed similar trends but different magnitudes for each mixture. The compressive strength of AAS mortar was enhanced significantly at 7 days, but then the rate of compressive strength increased less rapidly. Regardless of LBD addition, mortar mixes with 20% OPC had higher compressive strength values, compared with 10% OPC mortar mixes. Similar results were observed by Yang et al. [28]

The incorporation of LBD in AAS at a GGBFS replacement ratio of 10% had a significant effect on the compressive strength for all different ages, regardless of OPC content. For example, C20S70L10 showed the highest compressive strength value of 52.4 Mpa (range 52.1 Mpa to 52.7 Mpa) at 28 days of curing, which is 31.7% greater than C20S80 without LBD. In addition, C10S80L10 had a compressive strength value of 49.05 Mpa (range 48.7 Mpa to 49.4 Mpa) at 28 days of curing, which is approximately 60% greater than C10S90. Although mortar specimens containing 20% LBD showed lower compressive strength values than did those with 10% LBD, the C10S70L20 and C20S60L20 specimens had strength values that were 44% and 13.3% greater than those of the C10S90, and C20S80 specimens, respectively.

Hailong Ye et al. [41] found that the incorporation of dolomite induced porosity reduction and strength development, by enhancing the reaction degree of slag because of a filler effect. Machner et al. [47] showed similar enhancement of compressive strength results and attributed the compressive strength development to the use of dolomite through carbonate addition. The addition of dolomite induced an ettringite stabilization effect and the formation of carbonate AFm phases.

### 3.5. Flexural Strength

**Figure 8:**
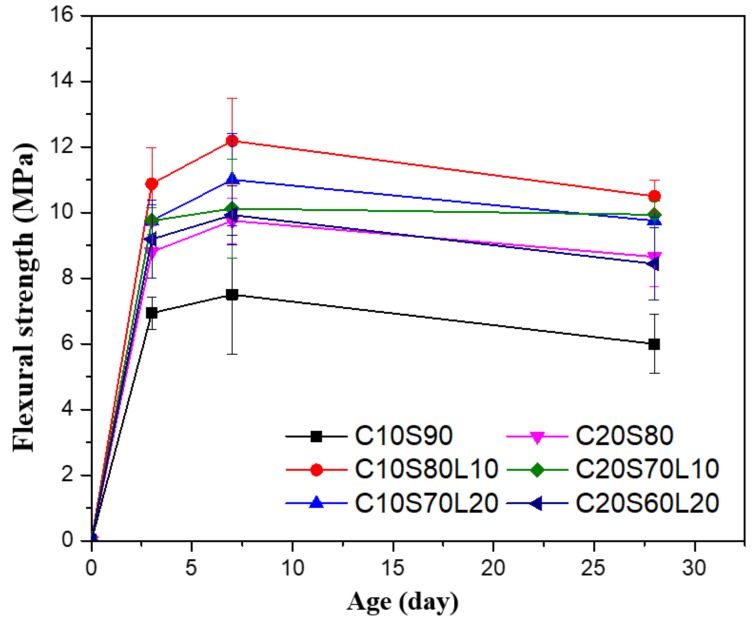
Flexural strength of alkali-activated slag mortar with different mixtures.

Figure 8 shows the influence of LBD incorporation on the flexural strength development of AAS mortar mixtures. Flexural strength showed a different tendency than did compressive strength, which increased steadily over time. In all mortar mixtures, flexural strength tended to increase until 7 days of curing, but decreased somewhat by 28 days of curing. The mixtures with 10% LBD, in AAS, showed higher flexural strength values, compared with 0% LBD mixtures for all curing ages. This is similar to compressive strength development. C10S80L10 specimens showed the highest flexural strengths at all curing ages: 10.88 Mpa (range 10.33 Mpa to 11.43 Mpa), 12.19 Mpa (range 11.54 Mpa to 12.84 Mpa), and 10.5 Mpa (range 10.25 Mpa to 10.75 Mpa) at 3 days, 7 days, and 28 days of curing. The tendency for reduced flexural strength after 7 days in an AAS system was also demonstrated in a previous study [42]. In addition, Nedeljković et al. [42] found that large deviations in flexural strength values were mainly due to microcracks. In AAS systems, Hubler et al. [48], Thomas et al. [49], and Collins and Sanjayan [50] observed microcracks both inside, and on the surface of, the AAS samples. With increasing hydration, the mixtures showed greater compressive strength regardless of the microcracks. However, the microcracks in specimens resulted from several factors; for example, surface drying shrinkage played a major role in reducing flexural strength. The drying shrinkage of AAS mortar is discussed in Section 3.6. Duxson et al. [51] proposed a different concept to demonstrate flexural strength behavior through the defect density of unreacted material. Duxson believed that an enhanced amount of unreacted material would increase the defect density. Thus, potential methods of failure would increase due to the increased defect density.

### 3.6. Drying Shrinkage of Mortar Specimens

**Figure 9:**
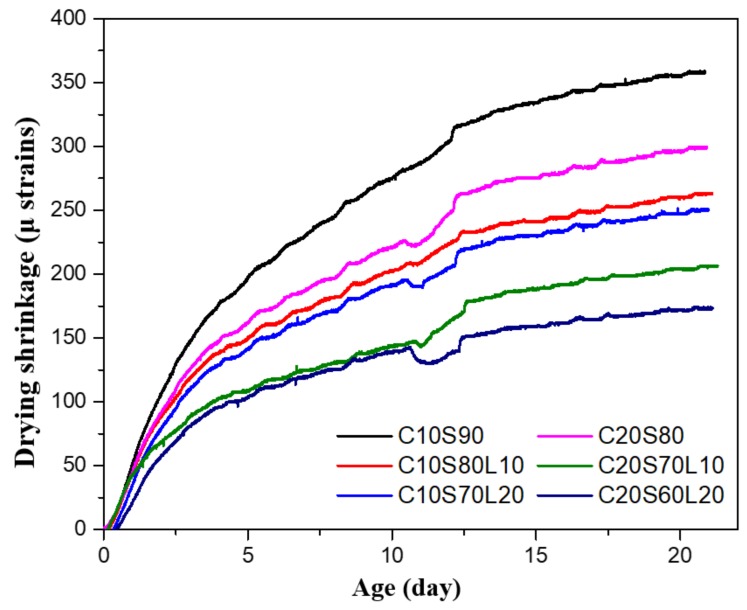
Drying shrinkage of alkali-activated slag mortar with different mixtures.

The drying shrinkage values of samples with different OPC and LBD content are shown in Figure 9. Regardless of LBD content, greater drying shrinkage was shown in all mixtures containing 10% OPC, compared with mixtures containing 20% OPC. Increased alkali-activated binder content improved the drying shrinkage. These trends were also reported in previous studies. Collins and Sanjayan [21] found that AAS had drying shrinkage values that were approximately three times greater than those of the OPC concrete. Aydin et al. [52] investigated the drying shrinkage of AAS with different hydration products. The common hydration products of AAS are C-S-H gels, which have low Ca/Si ratios (0.8–1.1), and lack of crystal phases, such as Ca(OH)_2_, leading to higher drying shrinkage. All specimens showed a similar trend, with higher LBD content being associated with a lower drying shrinkage value. After 3 weeks, the drying shrinkage values decreased by 26.3%, and 29.8% for C10S80L10, and C10S70L20, respectively, compared with C10S90 (which only consists of OPC and GGBFS). In addition, C20S70L10 and C20S60L20 had drying shrinkage values that were approximately 31% and 42% less than that of C20S80, respectively. These results are consistent with previous studies [53,54]. The decreased drying shrinkage associated with LBD could be attributed to the role of reactive MgO in LBD. The reactive MgO inside LBD can accelerate the hydration of the AAS system and fill the pores, so it helps to reduce the drying shrinkage. Fang et al. [55] and Jin et al. [56] showed that the use of reactive MgO in alkali-activated binder can reduce the drying shrinkage.

### 3.7. XRD Analysis

**Figure 10:**
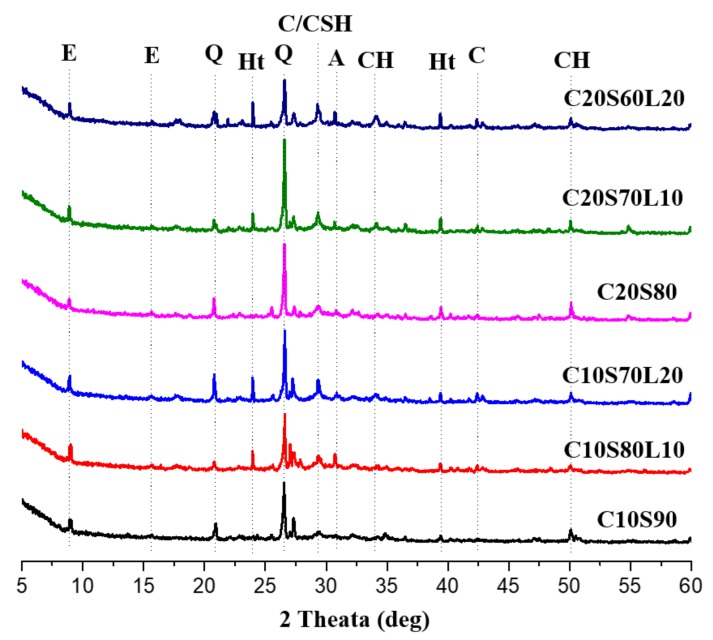
XRD patterns of AAS mixtures with different OPC and LBD content at 7 days. Key: E–ettringite Ca_6_Al_2_(SO_4_)_3_(OH)_12_•26H_2_O, Q–Quartz, Ht–hydrotalcite Mg_6_Al_2_CO_3_(OH)_16_•4H_2_O, C–Calcite, CSH–Calcium silicate hydrate, A–akermanite Ca_2_Mg(Si_2_O_7_), CH–Calcium hydroxide.

Figure 10 shows the XRD patterns of the alkali-activated slag mixtures, with different OPC and LBD content, at 7 days of curing. In sodium sulfate-activated slag mixtures, the main reacted products are ettringite and C-S-H [57]. Regardless of LBD content, ettringite and C-S-H could be identified in all AAS mixtures. Akermanite was identified due to the mineralogical properties of GGBFS. Calcium hydroxide peaks were measured in all mixtures, unlike typical sodium sulfate-activated slag mixtures, which can be attributed to the replacement of OPC content. The peak intensity of hydrotalcite in LBD-containing specimens was stronger than that of specimens without LBD. In addition, increasing LBD content was associated with greater hydrotalcite peak intensity. A high amount of reactive MgO in LBD can help to produce Mg-Al type layered double hydroxide. A distinct peak of C/CSH, which overlaps with C-S-H and calcite, could be seen in all mixtures; the intensity of these peaks was slightly enhanced with LBD content. This may be part of the reason for the increased heat evolution discussed in Section 3.3. LBD may accelerate the polymerization and hydration process of an AAS system and lead to the production of more C-S-H type gel. The increase in calcite peak intensity, due to the increase in LBD content, can be attributed to the large amount of calcite in LBD. The change in peak values from OPC content was not as evident as the apparent change in peak values from LBD content. It can be demonstrated that reactive CaO in LBD can produce the Ca(OH)_2_ with water and then the hydration proceeds with SiO_2_ in GGBFS to produce C-S-H gel, which leads to accelerate hydration procedure. Additionally, reactive MgO in LBD can form voluminous products, such as hydrotalcite and can produce dense microstructure [58,59].

### 3.8. TGA Analysis

**Figure 11:**
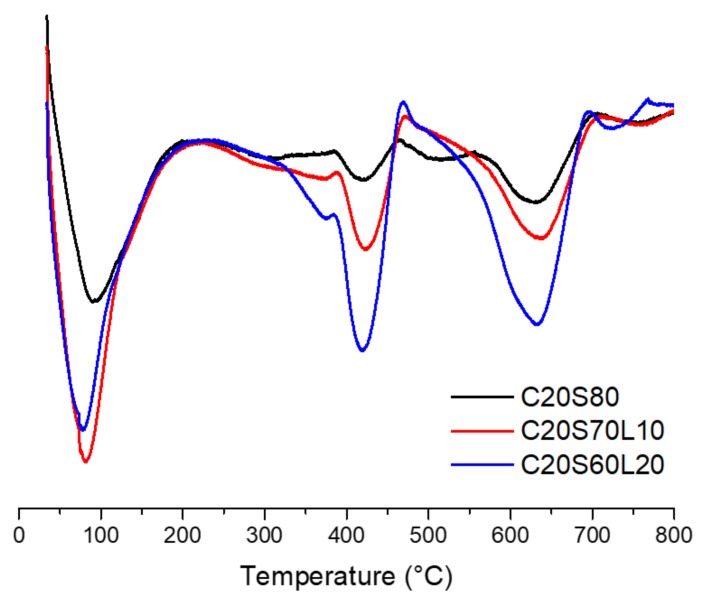
DTG curves of 20% OPC-based AAS mixtures with increasing LBD content.

Figure 11 shows the derivative thermogravimetric (DTG) analysis curves of 20% OPC-based AAS mixtures, with increasing LBD content. Regardless of LBD inclusion, all AAS mixtures showed mass loss peaks at four different temperature regions. The first mass loss peak is below 150 °C is related to the evaporation of free water [60], the dehydration of C-S-H [19], and ettringite [61]. The second mass loss peak is located between 350 and 400 °C, which can be attributed to the thermal dehydration of the hydrotalcite phase [62]. With increasing LBD content, a higher mass loss was indicated in the DTG curve of 20% OPC-based AAS mixtures because the higher amount of reactive MgO inside LBD helps to produce a hydrotalcite phase. The progressive mass loss, above approximately 400 °C, is related to the dehydration of a C-S-H type gel, such as C-A-S-H gel [60]. A higher amount of LBD in AAS mixtures shows a significant mass loss in this temperature region. When LBD was incorporated in an AAS system, LBD accelerated the polymerization process and assisted the hydration product in an AAS system, which is associated with cumulative heat release, as discussed in Section 3.3. The last mass loss peak is between 600 and 750 °C, which is attributed to the thermal decomposition of a carbonate product, such as calcite. In the DTG curve between 600 and 750 °C, the biggest mass loss peak is found in C20S60L20, followed by C20S70L10 and C20L80.

**Figure 12:**
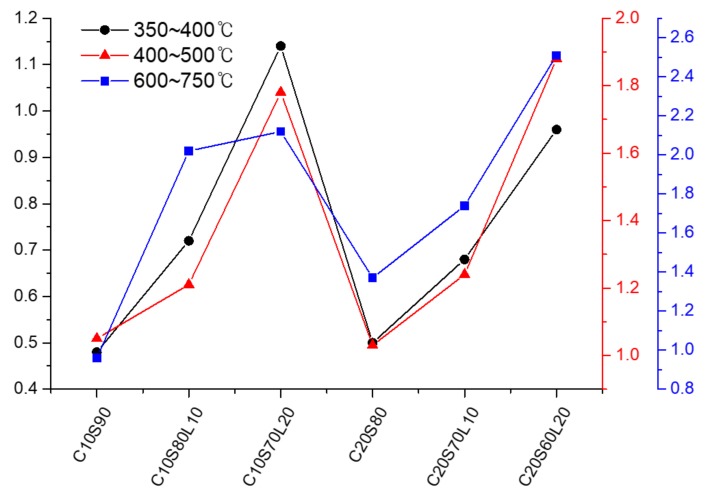
Mass loss fractions at different temperature ranges of AAS mixtures as a function of LBD.

LBD has more carbonate than OPC and GGBFS. Therefore, LBD in an AAS system might play a role in the formation of carbonate salt, due to the large amount of reactive CaO, MgO, and CO_3_^2-^, compared with GGBFS. Figure 12 shows that the mass loss fractions contribute to the hydrotalcite (350–400 °C), C-A-S-H gel (400–500 °C), and carbonate salt (600–750 °C). Regardless of OPC content, all AAS mixtures with 20% LBD had the greatest hydrotalcite, C-A-S-H gel, and carbonate salt mass loss fraction among the specimens that included LBD. Yang et al. [44] found that, using calcined dolomite consumes CO_3_^2-^ ions to produce the hydrotalcite phase, and then increases the alkalinity to accelerate C-A-S-H gel formation.

## 4. Conclusions

In this study, one-part sodium sulfate alkali-activated slag cement mixture was prepared using OPC, GGBFS, and LBD as binder materials, with solid sodium sulfate powder as the alkali activator. These experiments were conducted to investigate the effects of LBD incorporation on setting, strength, and drying shrinkage of an AAS system. From the results, the following conclusions can be made:

1. The replacement of up to 20% of GGBFS with LBD has minimal negative effects on the fluidity of AAS mixtures. As the replacement ratio increased to 20%, the fluidity reduction was approximately 10% to 17% compared to specimens without LBD based on 10% OPC specimens. The groups containing 20% OPC had slightly higher fluidity compared with groups containing 10% OPC.
2. Increasing LBD content tends to enhance the heat evolution of an AAS mixture. It also accelerates the polymerization and hydration process of an AAS system, which is due to the formation of calcium hydroxide from reactive CaO in LBD, resulting in a reduction of the initial, and final, setting times of AAS mixtures.
3. Replacing GGBFS with LBD has positive effects on compressive and flexural strength. Regardless of OPC content, LBD-containing specimens showed greater strength development than mixtures without LBD. Reactive CaO in LBD accelerate the hydration process in producing more C-S-H gel and reactive MgO in LBD produce voluminous hydration products to make dese microstructure. The highest compressive and flexural strength values were obtained from 10% LBD mixtures, followed by 20% and no-LBD content in both 10% and 20% OPC-based mixtures.
4. In terms of drying shrinkage, increasing the OPC content from 10% to 20% causes a reduction of GGBFS content, resulting in decreased shrinkage values. Replacing LBD with GGBFS can effectively reduce the drying shrinkage of sodium sulfate-activated AAS mixtures, regardless of OPC content.
5. The main hydration products of ettringite, C-S-H, and Ca(OH)_2_ were detected by XRD and TGA in all mixtures. Increasing amounts of C-S-H, hydrotalcite phase, and calcite were observed in LBD-containing specimens, which can be attributed to the reactive MgO, CaO, and calcite inside LBD.

**Further Research**

Future research should focus on the influence of LBD incorporation on microstructure and mechanical durability. Studies should also be conducted to investigate the microstructure, chloride penetration, and carbonation of AAS mixtures.

## Figures and Tables

**Table 1:** Chemical and physical properties of Ordinary Portland cement (OPC), ground granulated blast furnace slag (GGBFS), and light-burnt dolomite (LBD).

Composition	Weight (%)		
	OPC	GGBFS	LBD
SiO_2_	20.8	34.1	3.0
Al_2_O_3_	6.3	16.1	1.3
Fe_2_O_3_	3.2	0.4	0.6
CaO	62	42.3	52.2
MgO	3.3	4.1	25.3
SO_3_	2.2	2.5	0.4
Na_2_O	-	-	0.06
K_2_O	-	-	0.2
P_2_O_5_	-	-	0.03
TiO_2_	-	-	-
Loss on ignition	1.3	0.05	1.85
Specific surface area [cm^2^/g]	3200	4893	3038

**Table 2:** Mix composition of the specimens.

Specimen Code	OPC (wt%)	GGBFS (wt%)	LBD (wt%)	Sodium Sulfate	Sand	Water-to-Binder Ratio
C10S90	0.1	0.9	0	0.04	2	0.4
C10S80L10	0.1	0.8	0.1	0.04	2	0.4
C10S70L20	0.1	0.7	0.2	0.04	2	0.4
C20S80	0.2	0.8	0	0.04	2	0.4
C20S70L10	0.2	0.7	0.1	0.04	2	0.4
C20S60L20	0.2	0.6	0.2	0.04	2	0.4

## References

[1] Lee N., Jang J.G., Lee H.-K. (2014). Shrinkage characteristics of alkali-activated fly ash/slag paste and mortar at early ages. Cem. Concr. Compos..

[2] Duxson P., Fernández-Jiménez A., Provis J.L., Lukey G.C., Palomo A., Van Deventer J.S. (2007). Geopolymer technology: The current state of the art. J. Mater. Sci..

[3] Bernal S.A., Provis J.L. (2014). Durability of alkali-activated materials: Progress and perspectives. J. Am. Ceram. Soc..

[4] Duxson P., Provis J.L. (2008). Designing precursors for geopolymer cements. J. Am. Ceram. Soc..

[5] Luukkonen T., Abdollahnejad Z., Yliniemi J., Kinnunen P., Illikainen M. (2018). One-part alkali-activated materials: A review. Cem. Concr. Res..

[6] Mclellan B.C., Williams R.P., Lay J., Van Riessen A., Corder G.D. (2011). Costs and carbon emissions for geopolymer pastes in comparison to ordinary portland cement. J. Clean. Prod..

[7] Sturm P., Gluth G., Brouwers H., Kühne H.-C. (2016). Synthesizing one-part geopolymers from rice husk ash. Constr. Build. Mater..

[8] Abdollahnejad Z., Miraldo S., Pacheco-Torgal F., Aguiar J.B. (2017). Cost-efficient one-part alkali-activated mortars with low global warming potential for floor heating systems applications. Eur. J. Environ. Civ. Eng..

[9] Hajimohammadi A., Ngo T., Mendis P., Nguyen T., Kashani A., Van Deventer J.S. (2017). Pore characteristics in one-part mix geopolymers foamed by H_2_O_2_: The impact of mix design. Mater. Des..

[10] Provis J.L., Palomo A., Shi C. (2015). Advances in understanding alkali-activated materials. Cem. Concr. Res..

[11] Gebregziabiher B.S., Thomas R.J., Peethamparan S. (2016). Temperature and activator effect on early-age reaction kinetics of alkali-activated slag binders. Constr. Build. Mater..

[12] Roy D.M. (1999). Alkali-activated cements opportunities and challenges. Cem. Concr. Res..

[13] Shi C., Roy D., Krivenko P. (2003). Alkali-Activated Cements and Concretes.

[14] Pacheco-Torgal F., Labrincha J., Leonelli C., Palomo A., Chindaprasit P. (2014). Handbook of Alkali-Activated Cements, Mortars and Concretes.

[15] Pacheco-Torgal F., Castro-Gomes J., Jalali S. (2008). Alkali-activated binders: A review: Part 1. Historical background, terminology, reaction mechanisms and hydration products. Constr. Build. Mater..

[16] Wang S.-D., Scrivener K.L. (1995). Hydration products of alkali activated slag cement. Cem. Concr. Res..

[17] Lecomte I., Henrist C., Liegeois M., Maseri F., Rulmont A., Cloots R. (2006). (Micro)-structural comparison between geopolymers, alkali-activated slag cement and Portland cement. J. Eur. Ceram. Soc..

[18] Wang S.-D., Pu X.-C., Scrivener K., Pratt P. (1995). Alkali-activated slag cement and concrete: A review of properties and problems. Adv. Cem. Res..

[19] Taylor H.F. (1997). Cement Chemistry.

[20] Collins F., Sanjayan J.G. (1999). Strength and shrinkage properties of alkali-activated slag concrete placed into a large column. Cem. Concr. Res..

[21] Collins F., Sanjayan J.G. (2000). Effect of pore size distribution on drying shrinking of alkali-activated slag concrete. Cem. Concr. Res..

[22] Thomas R., Lezama D., Peethamparan S. (2017). On drying shrinkage in alkali-activated concrete: Improving dimensional stability by aging or heat-curing. Cem. Concr. Res..

[23] Ye H., Radlińska A. (2016). Shrinkage mechanisms of alkali-activated slag. Cem. Concr. Res..

[24] Ye H., Cartwright C., Rajabipour F., Radlińska A. (2017). Understanding the drying shrinkage performance of alkali-activated slag mortars. Cem. Concr. Compos..

[25] Neto A.A.M., Cincotto M.A., Repette W. (2008). Drying and autogenous shrinkage of pastes and mortars with activated slag cement. Cem. Concr. Res..

[26] Galı S., Ayora C., Alfonso P., Tauler E., Labrador M. (2001). Kinetics of dolomite–portlandite reaction: Application to Portland cement concrete. Cem. Concr. Res..

[27] GarcıíA E., Alfonso P., Labrador M., Galıí S. (2003). Dedolomitization in different alkaline media: Application to Portland cement paste. Cem. Concr. Res..

[28] Yang W.-H., Ryu D.-W., Park D.-C., Kim W.-J., Seo C.-H. (2014). A study of the effect of light-burnt dolomite on the hydration of alkali-activated Portland blast-furnace slag cement. Constr. Build. Mater..

[29] Choi S.-W., Jang B.-S., Kim J.-H., Lee K.-M. (2014). Durability characteristics of fly ash concrete containing lightly-burnt MgO. Constr. Build. Mater..

[30] ASTM International (2017). C150/C150M-17, Standard Specification for Portland Cement.

[31] ASTM International (2008). ASTM C109-Standard Test Method for Compressive Strength of Hydraulic Cement Mortars.

[32] ASTM International (2002). 348. Standard Test Method for Flexural Strength of Hydraulic-Cement Mortars.

[33] ASTM International (1998). C230/C230m. Standard Specification for Flow Table for Use in Tests of Hydraulic Cement.

[34] ASTM International (2001). 1437-01. Standard Test Method for Flow of Hydraulic Cement Mortar.

[35] ASTM International (2003). 191. Standard Test Method for Time of Setting of Hydraulic Cement by Vicat Needle.

[36] ASTM International (2002). 348-02: Standard Test Method for Flexural Strength and Modulus of Hydraulic Cement Mortars.

[37] Moon H., Ramanathan S., Suraneni P., Shon C.-S., Lee C.-J., Chung C.-W. (2018). Revisiting the effect of slag in reducing heat of hydration in concrete in comparison to other supplementary cementitious materials. Materials.

[38] ASTM International (2013). C490/C490M. Standard Practice for Use of Apparatus for the Determination of Length Change of Hardened Cement Paste, Mortar, and Concrete.

[39] Deb P.S., Nath P., Sarker P.K. (2014). The effects of ground granulated blast-furnace slag blending with fly ash and activator content on the workability and strength properties of geopolymer concrete cured at ambient temperature. Mater. Des. (1980–2015).

[40] Nath P., Sarker P.K. (2014). Effect of GGBFS on setting, workability and early strength properties of fly ash geopolymer concrete cured in ambient condition. Constr. Build. Mater..

[41] Ye H., Fu C., Yang G. (2019). Influence of dolomite on the properties and microstructure of alkali-activated slag with and without pulverized fly ash. Cem. Concr. Compos..

[42] Nedeljković M., Li Z., Ye G. (2018). Setting, strength, and autogenous shrinkage of alkali-activated fly ash and slag pastes: Effect of slag content. Materials.

[43] Bernal S.A., De Gutierrez R.M., Provis J.L., Rose V. (2010). Effect of silicate modulus and metakaolin incorporation on the carbonation of alkali silicate-activated slags. Cem. Concr. Res..

[44] Yang T., Zhang Z., Zhu H., Zhang W., Gao Y., Zhang X., Wu Q. (2019). Effects of calcined dolomite addition on reaction kinetics of one-part sodium carbonate-activated slag cements. Constr. Build. Mater..

[45] Zhang Z., Wang H., Provis J.L., Bullen F., Reid A., Zhu Y. (2012). Quantitative kinetic and structural analysis of geopolymers. Part 1. The activation of metakaolin with sodium hydroxide. Thermochim. Acta.

[46] Mehta P.K., Monteiro P.J. (2017). Concrete Microstructure, Properties and Materials.

[47] Machner A., Zajac M., Haha M.B., Kjellsen K.O., Geiker M.R., De Weerdt K. (2017). Portland metakaolin cement containing dolomite or limestone–Similarities and differences in phase assemblage and compressive strength. Constr. Build. Mater..

[48] Hubler M.H., Thomas J.J., Jennings H.M. (2011). Influence of nucleation seeding on the hydration kinetics and compressive strength of alkali activated slag paste. Cem. Concr. Res..

[49] Thomas J.J., Allen A.J., Jennings H.M. (2012). Density and water content of nanoscale solid C–S–H formed in alkali-activated slag (AAS) paste and implications for chemical shrinkage. Cem. Concr. Res..

[50] Collins F., Sanjayan J. (2001). Microcracking and strength development of alkali activated slag concrete. Cem. Concr. Compos..

[51] Duxson P., Provis J.L., Lukey G.C., Mallicoat S.W., Kriven W.M., Van Deventer J.S. (2005). Understanding the relationship between geopolymer composition, microstructure and mechanical properties. Colloids Surf. A: Physicochem. Eng. Asp..

[52] Aydın S., Baradan B. (2012). Mechanical and microstructural properties of heat cured alkali-activated slag mortars. Mater. Des..

[53] Mo L., Deng M., Tang M. (2010). Effects of calcination condition on expansion property of MgO-type expansive agent used in cement-based materials. Cem. Concr. Res..

[54] Cao F., Miao M., Yan P. (2018). Effects of reactivity of MgO expansive agent on its performance in cement-based materials and an improvement of the evaluating method of MEA reactivity. Constr. Build. Mater..

[55] Fang Y.H., Gu Y.M., Kang Q.B. (2011). Effect of fly ash, MgO and curing solution on the chemical shrinkage of alkali-activated slag cement. Advanced Materials Research.

[56] Jin F., Gu K., Al-Tabbaa A. (2015). Strength and hydration properties of reactive MgO-activated ground granulated blastfurnace slag paste. Cem. Concr. Compos..

[57] Rashad A., Bai Y., Basheer P., Milestone N., Collier N. (2013). Hydration and properties of sodium sulfate activated slag. Cem. Concr. Compos..

[58] Djayaprabha H.S., Chang T.-P., Shih J.-Y., Chen C.-T. (2017). Mechanical properties and microstructural analysis of slag based cementitious binder with calcined dolomite as an activator. Constr. Build. Mater..

[59] Gu K., Jin F., Al-Tabbaa A., Shi B. (2014). Activation of ground granulated blast furnace slag by using calcined dolomite. Constr. Build. Mater..

[60] Rivera O., Long W., Weiss C., Moser R., Williams B., Torres-Cancel K., Gore E., Allison P. (2016). Effect of elevated temperature on alkali-activated geopolymeric binders compared to portland cement-based binders. Cem. Concr. Res..

[61] Greene K.T. (1960). Early hydration reactions of Portland cement. Proc. Fourth Int. Congr. Chem. Cem..

[62] Rey F., Fornés V., Rojo J.M. (1992). Thermal decomposition of hydrotalcites. An infrared and nuclear magnetic resonance spectroscopic study. J. Chem. Soc. Faraday Trans..

